# Ascites-derived ALDH+CD44+ tumour cell subsets endow stemness, metastasis and metabolic switch via PDK4-mediated STAT3/AKT/NF-κB/IL-8 signalling in ovarian cancer

**DOI:** 10.1038/s41416-020-0865-z

**Published:** 2020-05-11

**Authors:** Yu-Xin Jiang, Michelle Kwan-Yee Siu, Jing-Jing Wang, Xue-Tang Mo, Thomas Ho-Yin Leung, David Wai Chan, Annie Nga-Yin Cheung, Hextan Yuen-Sheung Ngan, Karen Kar-Loen Chan

**Affiliations:** 1 Department of Obstetrics and Gynaecology, LKS Faculty of Medicine, The University of Hong Kong, Pokfulam, Hong Kong; 2 Department of Pathology, LKS Faculty of Medicine, The University of Hong Kong, Pokfulam, Hong Kong

**Keywords:** Ovarian cancer, Cancer stem cells

## Abstract

**Background:**

Ovarian cancer is characterised by frequent recurrence due to persistent presence of residual cancer stem cells (CSCs). Here, we identify and characterise tumour subsets from ascites-derived tumour cells with stemness, metastasis and metabolic switch properties and to delineate the involvement of pyruvate dehydrogenase kinase 4 (PDK4) in such process.

**Methods:**

Ovarian cancer cells/cell lines derived from ascites were used for tumourspheres/ALDH+CD44+ subset isolation. The functional roles and downstream signalling of PDK4 were explored. Its association with clinical outcome of ovarian cancer was analysed.

**Results:**

We demonstrated enhanced CSC characteristics of tumour cells derived from ovarian cancer ascites, concomitant with ALDH and CD44 subset enrichment and high PDK4 expression, compared to primary tumours. We further showed tumourspheres/ALDH+CD44+ subsets from ascites-derived tumour cells/cell lines with CSC properties and enhanced glycolysis. Clinically, PDK4 expression was correlated with aggressive features. Notably, blockade of PDK4 in tumourspheres/ALDH+CD44+ subsets led to inhibition of CSC characteristics, glycolysis and activation of STAT3/AKT/NF-κB/IL-8 (signal transducer and activator of transcription 3/protein kinases B/nuclear factor-κB/interleukin-8) signalling. Conversely, overexpression of PDK4 in ALDH−CD44– subsets exerted the opposite effects.

**Conclusion:**

Ascites-derived ALDH+CD44+ tumour cell subsets endow stemness, metastatic and metabolic switch properties via PDK4-mediated STAT3/AKT/NF-κB/IL-8 signalling, suggesting PDK4 as a viable therapeutic molecular target for ovarian cancer management.

## Background

Ovarian cancer is one of the most lethal gynaecological malignancies worldwide. Cancer stem-like cells (CSCs) represent a small subpopulation of tumour cells with stem-like properties that are responsible for tumour growth, metastasis, chemoresistance and recurrence,^1^ leading to poor prognosis of ovarian cancer patients. Effective targeting of this subset of cells may therefore eventually aid in control of the disease. Accumulating studies have identified and characterised a self-renewing subpopulation of CSCs displaying stem-like properties in ovarian cancer,^2^ although the associated regulatory mechanisms remain unknown at present.

Owing to their location as intra-abdominal tumours, ovarian cancer cells exfoliated from the primary ovarian tumour floating in peritoneal fluid are prone to forming three-dimensional (3D) multicellular aggregates (spheroids), which serve as a vehicle for tumour cell dissemination within the peritoneal cavity.^3^ Patients with advanced ovarian cancer are often diagnosed with peritoneal fluid accumulation (also known as ascites fluid). Ovarian cancer spheroids derived from ascites and ovarian cancer cell lines are produced over successive generations, consistent with the known sphere-forming and self-renewal characteristics of stem cells.^4^ These spheroids display upregulation of CSC markers and similar properties to CSC, such as assisting metastasis development and tumour escape from chemotherapy, implicating their enrichment in cells with CSC characteristics.^4^ Increasing evidence has been obtained on subpopulations of ascites-derived cells with CSC markers and properties, including sphere formation, chemoresistance, tumour initiation and serial tumour propagation.^2,5^ However, the underlying mechanisms are yet to be established.

High lactate production and low glucose oxidation, regardless of oxygen availability, a phenomenon known as aerobic glycolysis (Warburg effect), are commonly observed in various cancers.^6^ Recent studies have shown that CSC metabolism is similar to that of healthy tissue stem cells, which prefer to rely on aerobic glycolysis for energy supply with reduced mitochondrial activity, compared to differentiated cells.^7^ CSCs favour glycolysis rather than oxidative phosphorylation (OXPHOS) in multiple cancer types, including liver, breast and colorectal tumours.^8^ Exploitation of the metabolic characteristics of CSCs, especially the properties that are not expressed by differentiated cancer cells, may facilitate the development of novel therapeutic strategies for ovarian cancer.

Pyruvate dehydrogenase kinase 4 (PDK4) isoform is a gatekeeping enzyme that negatively phosphorylates pyruvate dehydrogenase, leading to conversion of pyruvate to lactate in the cytoplasm instead of further oxidation in mitochondria (i.e. OXPHOS).^9^ PDK4 is mostly detected in the skeletal muscle and heart,^10^ and its overexpression has been reported in glioblastoma, breast^11^ and colon^12^ cancers. A recent study revealed that PDK4 induces tumour growth through regulating the KRAS pathway in lung and colorectal tumours.^13^

Here, we compared the CSC properties of primary ovarian tumour and ascites-derived tumour cells, and subsequently analysed CSC properties, metabolic switches and PDK4 expression in spheroid cells (tumourspheres) and in the ALDH+CD44+ subset derived from ascites and ascites-originating ovarian cancer cell lines versus monolayer cells and the ALDH−CD44− subset. The clinical significance of PDK4 in ovarian cancer was further determined. Functional characterisation was performed and the downstream signalling pathway of PDK4 potentially contributing to CSC properties in tumourspheres/ALDH+CD44+ subsets derived from ascites or ascites-originating cell lines was delineated. Ascites-derived tumour cells exhibited enhanced CSC characteristics and PDK4 overexpression. Moreover, in vitro and in vivo experiments showed increased CSC properties and enhanced glycolysis of cell subpopulations isolated based on tumourspheres formation/CSC markers ALDH and CD44 from ascites and ovarian cancer cell lines, compared to monolayer cells/ALDH−CD44− subsets. Inhibition of PDK4 via the small interfering RNA (siRNA) approach or treatment with a pan-PDK inhibitor, dichloroacetate (DCA), led to suppression of cancer cell stemness and tumour growth through the STAT3/AKT/NF-κB/IL-8 (signal transducer and activator of transcription 3/protein kinases B/nuclear factor-κB/interleukin-8) signalling pathway. Conversely, overexpressed PDK4 in the ALDH−CD44− subpopulation exerted the opposite effects. Our data collectively supported the potential utility of PDK4 as a therapeutic target for management of ovarian cancer driven by ascites-derived CSCs.

## Materials and methods

Clinical samples and cell lines, immunohistochemistry (IHC), real-time PCR (qPCR), immunoblot analysis, enzyme-linked immunosorbent assay (ELISA), transwell migration and invasion assays, clonogenic assay, sphere formation assay, metabolic assays and the Cancer Genome Atlas Dataset (TCGA) and Gene Expression Profiling Interactive Analysis (GEPIA) are described in Supplementary Materials and methods.

### Tumourspheres culture

Cells were seeded as single-cell suspensions in 6-well ultra-low attachment plates at a density of 2000–5000 cells/well with serum-free DMEM/F12 (Dulbecco’s modified Eagle’s medium/F12) (1:1) (Gibco, MD, USA) supplemented with 10 ng/mL basic fibroblast growth factor (Sigma-Aldrich, MO, USA), 20 ng/mL human epidermal growth factor (Sigma-Aldrich) and 5 μg/mL insulin. Fresh medium was added to each well every 2 days without withdrawing the old medium.

### Flow cytometry and fluorescence-activated cell sorting

Anti-CD44-APC, anti-IgG-APC (Miltenyi Biotec, Germany) and ALDEFLUOR^TM^ kits (StemCell Technologies, Canada) were employed for flow cytometry analysis according to the manufacturer’s instructions. ALDH and CD44 double-positive and double-negative cells were isolated via fluorescence-activated cell sorting (FACS). Cells suspended in ALDEFLUOR™ Assay Buffer were incubated with ALDEFLUOR™ Reagent for 30 min at 37 °C. After centrifugation at 300 × *g* for 10 min, supernatant fractions were removed. Flow Buffer was added to resuspend cell pellets along with FcR Blocking Reagent and APC-conjugated CD44 antibody. The mixture was incubated for 30 min at 4 °C in the dark. Sorted cells were collected in complete medium with 2% P/S and incubated at 4 °C before seeding into culture plates.

### siRNA transfection

PDK4 and control siRNA were commercially obtained (Life Technologies, Canada) and introduced into cancer cells using SilentFect (Bio-Rad, Canada). At 48 h after transfection, cells were collected for use in subsequent assays.

### Transient overexpression of PDK4

PDK4 or control vector plasmids (GFP-tagged; OriGene, MD, USA) were transfected into cancer cells using Lipofectamine 3000 (Invitrogen, Canada) for 48 h and the cells plated for subsequent assays.

### Treatment with cisplatin, DCA, stattic and DMAPT

For cisplatin experiments, ALDH+CD44+ and ALDH−CD44− SKOV3 cells were plated 12 h before treatment with cisplatin (0 and 20 μM; Sigma-Aldrich) or control vehicle (ddH_2_O) for 48 h. For DCA or stattic experiments, ALDH+CD44+ SKOV3 cells were plated 12 h before treatment with DCA (0, 5 and 10 mM; Sigma-Aldrich), stattic (0, 7.5 and 10 μM; Sigma-Aldrich) or control vehicles (ddH_2_O for DCA and dimethyl sulfoxide (DMSO) for stattic) for 48 h. For co-treatment experiments, ALDH+CD44+ SKOV3 cells were plated 12 h before simultaneous treatment with cisplatin (0, 10 and 20 μM) and DCA (10 mM) or control vehicle (ddH_2_O) for 48 h. For dimethylaminoparthenolide (DMAPT) experiments, (i) ALDH+CD44+ SKOV3 cells were plated 12 h before treatment with DMAPT (0 and 1 μM; Abcam, UK) or control vehicle (DMSO) for 48 h and (ii) ALDH-CD44− SKOV3 cells transiently transfected with control vector or PDK4 were treated with DMAPT (0 and 1 μM) or control vehicle after 24 h of transfection for 48 h.

### Treatment with IL-8 and CXCR1 neutralising antibody

Following transfection with PDK4 or control vector plasmid for 24 h, ALDH−CD44− SKOV3 cells were treated with mouse IgG (2 μg/mL), neutralising antibody against CXCR1 (2 μg/mL) or recombinant IL-8 (100 ng/mL) (R&D) for 48 h.

### In vivo tumorigenicity analysis via subcutaneous implantation, limiting dilution assay and serial transplantation

Animal experiments were conducted following protocols approved by the Committee of the Use of Live Animals in Teaching and Research (CULATR) and were carried out under an approved CULATR licence (No. 4598-18). BALB/c nude mice (7–8 weeks), which are athymic with T cell deficiency were used for this study and kept in isolated cages in a sterile area supplied with constant air at 25 °C by Laboratory Animal Unit (LAU) of the University of Hong Kong. The life activity and health situation of the nude mice were monitored by LAU stuff. Sorted ALDH+CD44+ and ALDH−CD44− cells were harvested and suspended in a mixture containing RPMI medium and Matrigel (High Concentration, Corning, #354248). Different dilution (3 × 10^5^, 10 × 10^3^, 5 × 10^3^, 2.5 × 10^3^ cells) of ALDH+CD44+ or ALDH−CD44− cells were subcutaneously injected into flanks of mice in different groups. Four to six mice were randomised to each group, and tumour incidence and latency were recorded. The estimated frequency of CSCs was calculated using the Extreme Limiting Dilution Analysis software (http://bioinf.wehi.edu.au/software/elda/). Experimental mice were maintained for 2 months and were killed with 150 mg/kg pentobarbital, followed by tumour dissection and recording of tumour sizes. To confirm the lack of tumour formation in mice exhibiting no tumour nodules, injection sites were opened and observed. Isolated tumours were collected and dissociated into single cells. The harvested cells were counted and subsequently injected into secondary recipient mice.

To determine the effects of DCA on tumour initiation in vivo, FACS-sorted ALDH+CD44+ SKOV3 cell subpopulations were treated with DCA (10 mM) or control vehicle (ddH_2_O) for 3 days and harvested. Serial dilutions (3 × 10^5^, 10 × 10^3^, 5 × 10^3^, 2.5 × 10^3^ cells) of treated cells were injected into mice, and tumour incidence and latency were recorded. Tumour-initiating frequency was calculated as above and tumour sizes of 3 ×1 0^5^ cells measured every 3–5 days.

### Statistical analysis

Values are presented as the means ± SEM. The GraphPad Prism 7.0 software was applied to plot data with a two-tailed *t* test or one-way analysis of variance. *P* values <0.05 were regarded as statistically significant (*p < 0.05 and **p < 0.01). To determine the prognostic effects of PDK4 on ovarian cancer, Kaplan–Meier Plotter, a free online tool (www.kmplot.com), was utilised. Hazard ratios and log-rank *p* values were calculated automatically based on the optimal cut-off value auto-selected with the software.

## Results

### Ascites-derived tumour cells exhibit CSC properties and express high levels of PDK4

**Fig. 1:**
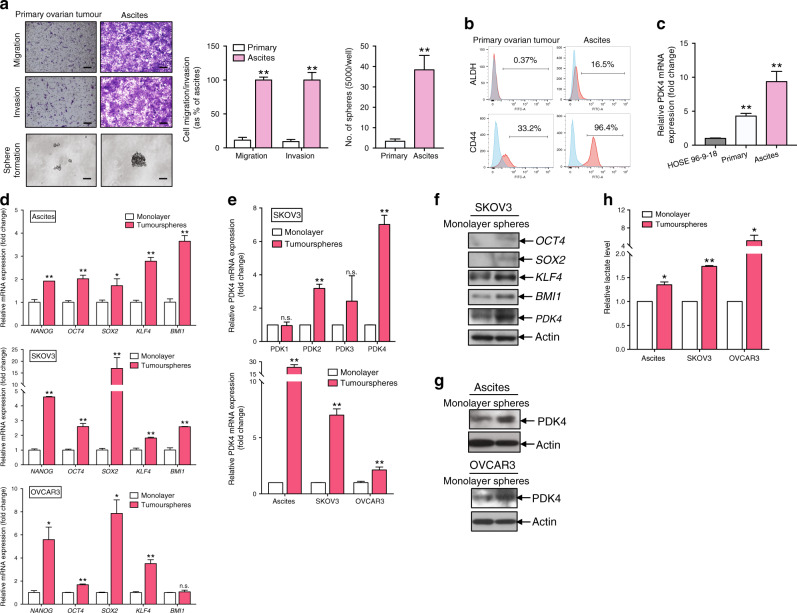
Ascites-derived tumour cells and tumourspheres show CSC properties and express higher levels of PDK4. **a** Transwell migration/invasion and sphere formation assays of cancer cells derived from primary ovarian tumours and ascites. (Left) Representative images of migrating or invading cells (scale bar, 100 μΜ) and sphere formation (scale bar, 50 μΜ). (Middle) Cell migration and invasion are presented as a percentage of ascites. (Right) Quantification of cells forming tumourspheres. **b** Flow cytometry analysis of ALDH and CD44 in single cells isolated from primary ovarian tumour or ascites. **c** Relative PDK4 expression in HOSE 96-9-18 and cancer cells derived from primary ovarian tumours and ascites determined via qPCR. **d** qPCR analysis of relative stemness genes, *NANOG*, *OCT4*, *SOX2*, *KLF4* and *BMI1*, of tumourspheres formed from ascites-derived tumour cells and ovarian cancer cell lines (SKOV3 and OVCAR3), compared to monolayer cells. **e** (Upper) Relative mRNA expression of PDK 1–4 in tumourspheres and monolayer cells isolated from SKOV3 cells determined using qPCR. (Lower) Relative PDK4 mRNA expression in tumourspheres and monolayer cells isolated from ascites, SKOV3 and OVCAR3 cells. **f** Immunoblotting showing protein expression of *OCT4*, *SOX2*, *KLF4*, *BMI1* and *PDK4* of tumourspheres and monolayer cells isolated from SKOV3 cells. **g** Immunoblotting showing PDK4 expression in tumourspheres and monolayer cells isolated from ascites cancer cells and OVCAR3 cells. **h** Relative lactate production of tumourspheres and monolayer cells isolated from ascites, SKOV3 and OVCAR3 cells after 24 h incubation assessed using Lactate Colorimetric Assay Kit II (*p < 0.05, **p < 0.01, results represent means ± SD from three independent experiments; n.s., not significant).

We isolated ovarian cancer cells from primary tumours and ascites samples. The epithelial nature of primary cells was confirmed based on positivity for the epithelial markers, CK7 and AE1/AE3, and negativity for the fibroblast marker, CD45 (Supplementary Fig. S1). Transwell migration and invasion assays revealed higher cell migration and invasion capabilities of ascites-derived tumour cells than primary ovarian tumour cells (Fig. 1a). In the sphere formation assay, larger and higher numbers of spheroids were observed from ascites-derived tumour cells relative to primary ovarian tumour cells (Fig. 1a). Additionally, higher ALDH and CD44 activities were detected in ascites-derived tumour cells than primary ovarian tumour cells (Fig. 1b). By qPCR analyses, significantly higher PDK4 expression in ascites-derived tumour cells, compared to primary ovarian tumour cells and the normal ovarian epithelial cell line, HOSE 96-9-18 (Fig. 1c). Our findings clearly suggested that ascites-derived tumour cells displayed CSC properties with increased PDK4 expression.

### Tumourspheres display CSC properties and express higher PDK4 levels than monolayer cells

Tumourspheres cultured in ultra-low attachment plates containing CSCs provide an efficient means to enrich CSCs in ovarian cancer.^14^ Primary tumoursphere cultures were established with ascites-derived tumour cells and ovarian cancer cell lines, SKOV3 and OVCAR3. qPCR analysis led to the identification of significantly upregulated messenger RNA (mRNA) of stemness genes in tumourspheres, compared with the bulk of parental cells cultured in monolayer conditions (Fig. 1d). Consistently, immunoblotting revealed higher protein expressions of stemness genes in tumourspheres relative to monolayer cells in SKOV3 (Fig. 1f). Among the four PDK isoforms, PDK2 and PDK4 levels were significantly higher in tumourspheres than monolayer cells in the SKOV3 group, with the greatest increase observed for PDK4 (Fig. 1e). Significant PDK4 mRNA expression was additionally detected in tumourspheres cultured from ascites-derived tumour cells and OVCAR3 (Fig. 1e). In addition, PDK4 protein expression was higher in tumourspheres from ascites-derived tumour cells (Fig. 1g) and ovarian cancer cell lines (Fig. 1f, g), as determined from immunoblot analyses. These findings suggested that tumourspheres derived from ascites-derived tumour cells and cell lines maintain CSC properties. Furthermore, enhanced lactate production in tumourspheres relative to monolayer cells was demonstrated by lactate assay (Fig. 1h).

### ALDH+CD44+ cells isolated from ascites-derived tumour cells show enhanced CSC properties and PDK4 expression

**Fig. 2:**
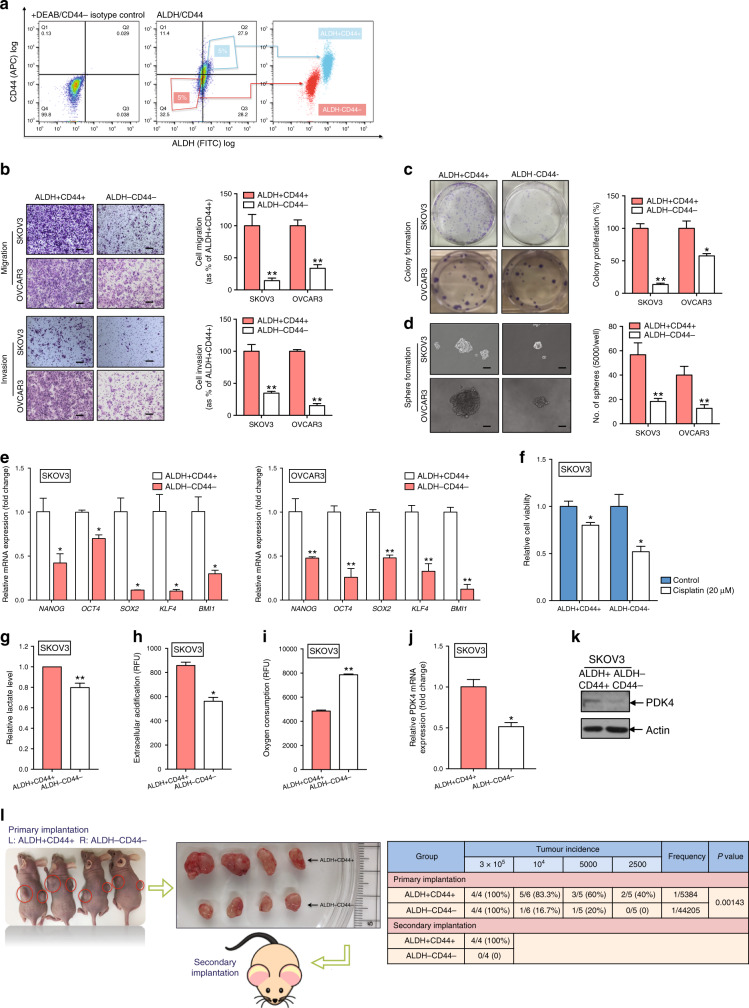
ALDH+CD44+ cells derived from ovarian cancer cells shows enhanced CSC properties and PDK4 expression. **a** FACS-mediated isolation of ALDH+CD44+ and ALDH−CD44− SKOV3 cells. (Left) Isotype control stained with DEAB and FITC IgG. (Middle) The top 5% cells most brightly stained for ALDH+CD44+ or bottom 5% with minimal staining for ALDH−CD44− cells were collected. (Right) Purity of ALDH and CD44 was confirmed via post sorting using flow cytometry. **b** Transwell migration/invasion (scale bar, 100 μΜ) and **c** clonogenic assays of ALDH+CD44+ and ALDH−CD44− cells isolated from SKOV3 and OVCAR3 cells were imaged and presented as a percentage of ALDH−CD44− groups. **d** Sphere formation assay of ALDH+CD44+ and ALDH−CD44− cells isolated from SKOV3 and OVCAR3 cells, followed by imaging (scale bar, 50 μΜ) and quantification of the number of cells that formed tumourspheres. **e** mRNA expression of relative stemness genes, *NANOG*, *OCT4*, *SOX2*, *KLF4*, and *BMI1*, in ALDH+CD44+ and ALDH−CD44− cells isolated from SKOV3 and OVCAR3 cells. **f** Relative cell viability of ALDH+CD44+ and ALDH−CD44− SKOV3 cells after treatment with or without 20 μM cisplatin for 48 h, determined with the XTT assay. **g** Relative lactate production of ALDH+CD44+ and ALDH−CD44− SKOV3 cells after 24 h incubation. **h** Extracellular acidification and **i** OCR assays of ALDH+CD44+ and ALDH−CD44− SKOV3 cells after 2 h incubation measured using the glycolysis and extracellular oxygen consumption assays, respectively (RFU: relative florescence units). Relative PDK4 **j** mRNA and **k** protein expression of ALDH+CD44+ and ALDH−CD44− SKOV3 cells via qPCR and immunoblotting, respectively. **l** (Left) Representative images of xenograft tumours resected from mice inoculated with ALDH+CD44+ and ALDH−CD44− SKOV3 cells (*n* = 4). Resected tumours were re-inoculated into the flanks of the second batch of mice. (Right) Summary of tumour incidence and estimated frequency of ALDH+CD44+ and ALDH−CD44− SKOV3 cells determined via in vivo limiting dilution and self-renewal assays (*p < 0.05, **p < 0.01, results are presented as means ± SD from three independent experiments).

In view of the increased ALDH and CD44 expression in ascites-derived tumour cells, FACS was employed to isolate ALDH+CD44+ and ALDH−CD44− subsets of SKOV3 and OVCAR3 cell groups. The top 5% cells showing highest staining levels for ALDH+CD44+ and bottom 5% cells with lowest staining for ALDH−CD44− were used in subsequent functional assays. The purity of the ALDH and CD44 cell subsets was confirmed via post-sorting using flow cytometry (Fig. 2a). Compared with the ALDH−CD44− subset, ALDH+CD44+ SKOV3 cells showed enhanced migration and invasion abilities (Fig. 2b). The clonogenic assay was employed to examine the activity of a single cell to develop into a large colony through clonal expansion, a sensitive indicator for CSCs.^15^ Notably, ALDH+CD44+ SKOV3 cells formed a greater number and larger-sized colonies than the ALDH−CD44− subset (Fig. 2c), along with an enhanced sphere-forming capacity (Fig. 2d). As expected, increased mRNA expression of stemness genes was detected in ALDH+CD44+ cancer cells, compared with the ALDH−CD44− subset (Fig. 2e). Accumulating evidence suggests that chemotherapy targets the bulk of tumour cells, whereas CSC subpopulations of tumours show a reduced response to chemotherapeutic drugs.^16^ Compared with the ALDH−CD44− subset, ALDH+CD44+ SKOV3 cells exhibited greater chemoresistance (Fig. 2f).

Next, we explored the differences in glycolytic metabolism between the two subsets. Significantly higher lactate production was observed in ALDH+CD44+, compared with the ALDH−CD44− SKOV3 subset (Fig. 2g). In the glycolysis assay, extracellular acidification rate reflecting lactic acid production through the glycolytic pathway was consistently upregulated in ALDH+CD44+ SKOV3 cells, compared with the ALDH−CD44− subset (Fig. 2h). Oxygen consumption rate (OCR), a sensitive indicator of the OXPHOS pathway, was lower in ALDH+CD44+ SKOV3 relative to the ALDH−CD44 subset (Fig. 2i). Moreover, PDK4 expression was significantly higher in ALDH+CD44+ SKOV3 cells at both mRNA (Fig. 2j) and protein (Fig. 2k) levels. Our data collectively indicated that the ALDH+CD44+ subset displayed a distinct metabolic profile with increased glycolytic influx and decreased OXPHOS activity.

Two significant CSC characteristics (tumour initiation and self-renewal) of ALDH+CD44+ cells were investigated using in vivo tumorigenesis and limiting dilution assays. In the in vivo tumorigenesis assay, mice in the ALDH+CD44+ group formed significantly larger tumours, compared to the ALDH−CD44− group (Fig. 2l). The in vivo limiting dilution assay involved injection of a series of cell dilutions into either the left or right flanks of nude mice. ALDH+CD44+ cells displayed enhanced tumour-initiating activity, as evident from the increased tumour incidence and significantly higher estimated tumour-initiating cell frequency. Tumours were collected, minced and dissociated for secondary implantation onto another batch of nude mice to assess the self-renewal capacity of ALDH+CD44+ cells. After 8 days, new tumours were detected in the flanks of all four mice in the ALDH+CD44+ group. However, no tumours were present in the secondary implantation sites of nude mice from the ALDH−CD44− group (Fig. 2l). Based on these findings, we conclude that dual positivity for ALDH and CD44 defines a compelling marker set for isolation of the CSC subpopulation of ovarian cancer.

### PDK4 is overexpressed in ovarian cancer and correlated with metastasis and poor prognosis

**Fig. 3:**
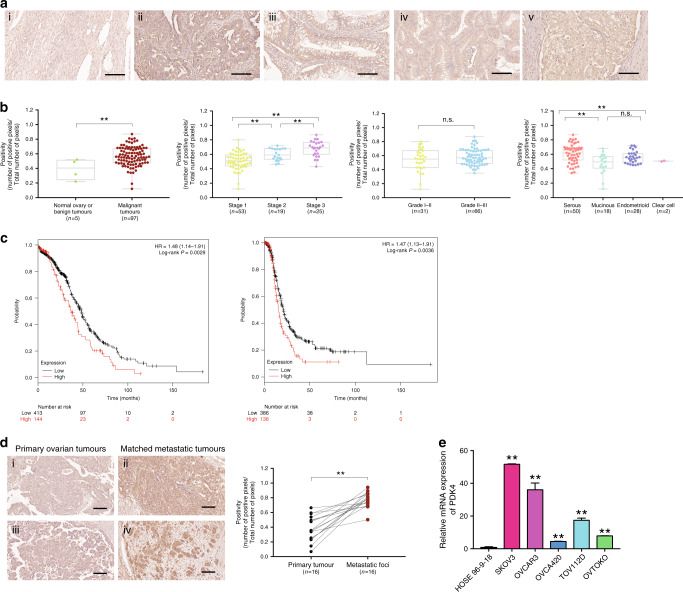
PDK4 is overexpressed in ovarian cancer and correlates with metastasis and poor prognosis. **a** Representative images (scale bar, 100 μΜ) of IHC staining for PDK4 expression in (i) normal ovarian tissue, (ii) serous, (iii) mucinous, (iv) endometrioid and (v) clear cell subtypes of tumour samples on ovarian cancer TMA (OVC1021, Biomax). **b** Correlation of PDK4 with clinicopathologic parameters in ovarian cancer. **c** (Left) Overall and (right) progression-free survival rates were analysed with the log-rank test for PDK^high/low^ groups of ovarian cancer patients obtained from TGCA using the Kaplan–Meier plotter. **d** (Left) Representative images (scale bar, 100 μΜ) of IHC staining for PDK4 expression in ovarian primary and matched metastatic tumours. (Right) Dot plot showing PDK4 positivity in primary and metastatic ovarian tumours. **e** PDK4 mRNA expression in normal human ovarian surface epithelial cell line (HOSE) and ovarian cancer cell lines as determined by qPCR (*p < 0.05, **p < 0.01; n.s., not significant).

PDK4 protein expression as determined from tissue microarray analyses was evaluated via IHC. Owing to predominant localisation in the cytoplasm, PDK4 staining was stronger in malignant tumour tissues relative to normal ovarian tissues/benign tumours (Fig. 3a) (*p* < 0.01). PDK4 protein expression was significantly higher in the advanced stages (stage 3) versus lower stages (stages 1 and 2). Serous ovarian tumours showed significantly higher expression of PDK4, compared to non-serous tumours, supporting a significant correlation between upregulation of PDK4 and ovarian carcinoma progression (Fig. 3b). In addition, we found that PDK4 mRNA was upregulated in ovarian cancer cell lines (SKOV3, OVCAR3, OVCA420, TOV112D and OVTOKO), compared to the normal human ovarian surface epithelial cell line, HOSE 96-9-18, by qPCR (Fig. 3e).

To assess the prognostic effect of PDK4 mRNA in clinical samples, we used the Kaplan–Meier Plotter with TCGA database in which 522 patients were grouped based on the optimal cut-off value auto-selected with the software. As shown from the survival curves (Fig. 3c), high PDK4 mRNA expression was correlated with poor overall (*p* = 0.0029) and progression-free (*p* = 0.0036) survival. The data indicated that PDK4, highly expressed in ovarian cancer tissues, was associated with poor patient outcome.

Moreover, significantly higher immunostaining of PDK4 was observed in metastatic foci than in the corresponding primary tumours from 16 pairs of FFPE tissue sections (Fig. 3d) (*p* < 0.01), supporting association of PDK4 with metastasis in ovarian cancer.

### PDK4 shifts ovarian CSC energy metabolism from OXPHOS to aerobic glycolysis and is crucial for CSC maintenance in vitro and in vivo

**Fig. 4:**
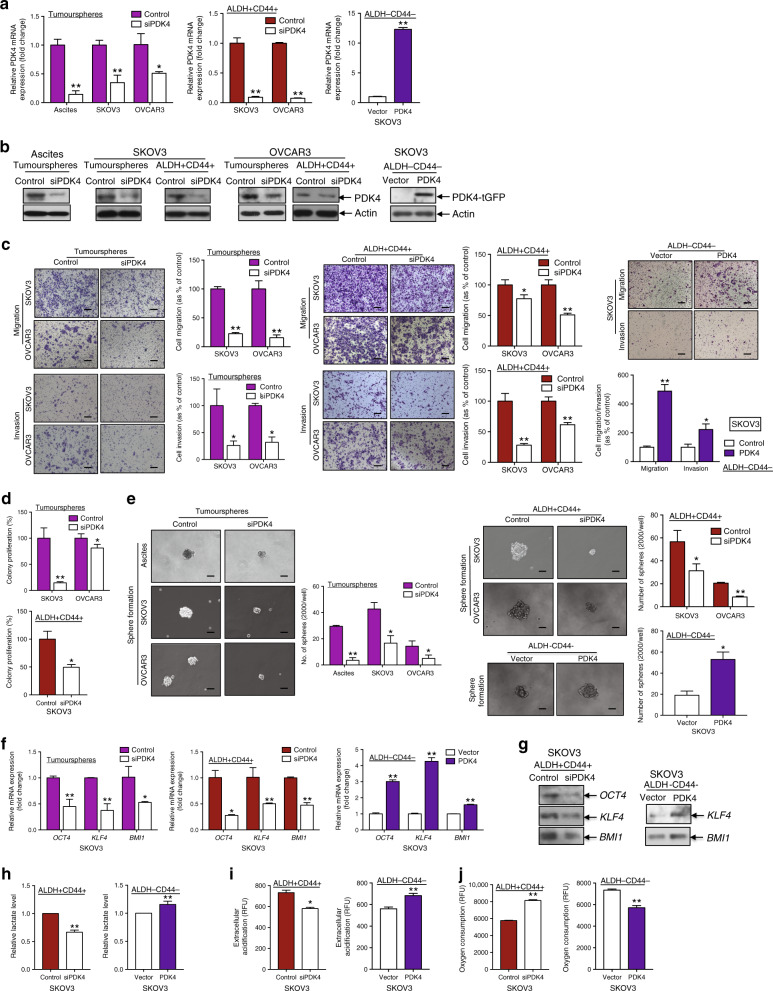
PDK4 shifts the mode of energy metabolism and is crucial for ovarian CSC maintenance. **a** Relative PDK4 mRNA and **b** protein expression in tumourspheres and ALDH+CD44+ cells transfected with control siRNA or siPDK4 and PDK4-overexpressing ALDH−CD44− cells, determined via qPCR and immunoblotting, respectively. **c** Images and quantification of data from transwell migration/invasion assays of (left) tumourspheres and (middle) ALDH+CD44+ cells transfected with control siRNA or siPDK4 and (right) PDK4-overexpressing ALDH−CD44− cells. Scale bar, 100 μΜ. **d** Clonogenic assays of (upper) tumourspheres and (lower) ALDH+CD44+ cells transfected with control siRNA or siPDK4 are quantified as a percentage of control groups. **e** Sphere formation assay of (left) tumourspheres and (right upper) ALDH+CD44+ cells transfected with control siRNA or siPDK4 and (right lower) PDK4-overexpressing ALDH−CD44− cells, followed by imaging (scale bar, 50 μΜ) and quantification of the number of cells that formed tumourspheres. **f** Relative mRNA and **g** protein expression of stemness genes *OCT4*, *KLF4* and *BMI1* in tumourspheres and ALDH+CD44+ cells transfected with control siRNA or siPDK4 and PDK4-overexpressing ALDH−CD44− cells determined via qPCR and immunoblotting, respectively. **h** Relative lactate production, **i** extracellular acidification and **j** OCR arrays of (left) PDK4-suppressed ALDH+CD44+ and (right) PDK4-overexpressing ALDH−CD44− SKOV3 cells after 24 h incubation (*p < 0.05, **p < 0.01, results represent means ± SD from three independent experiments).

To ascertain the correlation between PDK4 overexpression and altered CSC properties in ovarian cancer, we generated transient siPDK4-transfected tumourspheres/ALDH+CD44+ cells as well as ALDH−CD44− cells transiently overexpressing PDK4. qPCR (Fig. 4a) and immunoblot analyses (Fig. 4b) were employed to confirm transfection efficiency. PDK4 knockdown in tumourspheres/ALDH+CD44+ cells led to a significant decrease in migratory/invasive (Fig. 4c), clonogenic (Fig. 4d) and sphere-forming (Fig. 4e) capacities, while transient PDK4 overexpression exerted the opposite effects. Since cell death could affect the effects of PDK4 as presented above, we further evaluated the cell viability of tumourspheres and ALDH+CD44+ cells after PDK4 knockdown. PDK4 knockdown in tumourspheres and ALDH+CD44+ cells from SKOV3 and OVCAR3 did not show alteration in cell viability 1 day after incubation as determined using XTT and cell counting methods (Supplementary Fig. S2), suggesting that the effects on cell migration and invasion (determined after 1-day incubation) would not be related to cell death. We also found the cell viability of PDK4-depleted tumourspheres and ALDH+CD44+ cells from SKOV3 as 0.737–0.822-fold and OVCAR3 as 0.833–0.910-fold after 3 (SKOV3) and 5 (OVCAR3) days incubation compared to control as determined using XTT and cell counting methods (Supplementary Fig. S2), suggesting that the decreased sphere-forming ability would not result primarily from cell death.

We further used GEPIA to predict the correlations between PDK4 and five stemness genes (*NANOG*, *OCT4*, *SOX2*, *KLF4* and *BMI1*) that are highly expressed in tumourspheres and ALDH+CD44+ cells. Among the five genes, *KLF4* and *BMI1* mRNA levels were positively correlated with PDK4 (Supplementary Fig. S3). *NANOG*, *OCT4*, and *SOX2* displayed positive associations with PDK4, but not to a significant extent. Additionally, tumourspheres with suppression of PDK4 showed downregulated transcription of *OCT4*, *KLF4* and *BMI1* stemness genes (Fig. 4f). ALDH+CD44+ cancer cells transfected with siPDK4 exhibited lower expression of *OCT4*, *KLF4*, and *BMI1* genes at both mRNA (Fig. 4f) and protein levels (Fig. 4g). Consistent with these findings, introduction of PDK4 in ALDH−CD44− cells led to enhanced *OCT4*, *KLF4*, and *BMI1* mRNA (Fig. 4f), and KLF4 and BMI1 protein expression (Fig. 4g), supporting the conclusion that endogenous PDK4 regulated CSC properties in ovarian cancer.

Given that tumourspheres/ALDH+CD44+ cells appear to favour a metabolic switch from OXPHOS to glycolysis, we evaluated the role of PDK4 in the metabolic shift. ALDH+CD44+ SKOV3 cells with PDK4 knockdown showed decreased lactate production, compared with the control group (Fig. 4h), in keeping with decreased extracellular acidification (Fig. 4i) and increased OCR (Fig. 4j) exhibited by the PDK4-suppressed ALDH+CD44+ subset. Conversely, transient PDK4 overexpression led to increased lactate production (Fig. 4h) and extracellular acidification (Fig. 4i), as well as decreased OCR (Fig. 4j) in the ALDH−CD44− subset.

### DCA hinders ovarian CSC properties in vitro and in vivo

**Fig. 5:**
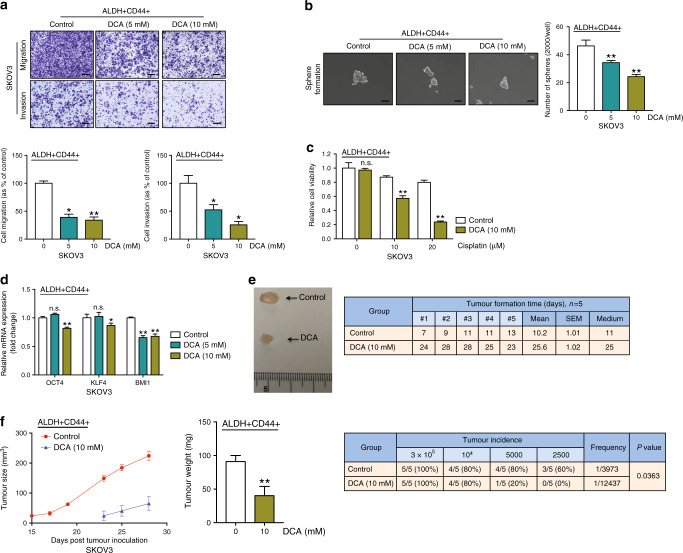
DCA hinders ovarian CSC properties in vitro and in vivo. **a** Transwell migration/invasion (scale bar, 100 μΜ) and **b** sphere formation assays (scale bar, 50 μΜ) of ALDH+CD44+ SKOV3 cells after treatment with vehicle and DCA (5 and 10 mM) for 48 h. **c** Relative cell viability of vehicle or 10 mM DCA-treated ALDH+CD44+ SKOV3 cells after treatment with or without 20 μM cisplatin for 48 h with the XTT assay. **d** qPCR analysis of mRNA expression of relative stemness genes, *OCT4*, *KLF4* and *BMI1*, of ALDH+CD44+ SKOV3 cells after treatment with DCA (5 and 10 mM). Following injection of 3 × 10^5^ ALDH+CD44+ SKOV3 cells pre-treated with control (*n* = 5) DCA (*n* = 5) into the right/left flanks of mice. **e** (Left) Representative images of xenograft tumours and (right) time of tumour formation (days) in the xenograft model were recorded. **f** (Left) Tumour size curves and (middle) weights of xenografts derived from control (*n* = 5) and DCA (*n* = 5)-pre-treated ALDH+CD44+ SKOV3 cells. (Right) The tumour initiation rate was recorded based on the in vivo limiting dilution assay (*p < 0.05, **p < 0.01, results represent means ± SD of three independent experiments; n.s., not significant).

Next, we determined whether DCA, a pharmacological PDK inhibitor,^17^ could mimic the effects of PDK4 silencing and block CSC properties in ovarian cancer cells. DCA is proposed to induce cell apoptosis in ovarian cancer by inducing a shift from anaerobic to aerobic metabolism.^18^ To determine the effects of DCA on CSC subpopulations in SKOV3 cells, ALDH+CD44+ cells were treated with DCA (0, 5 and 10 mM) and subjected to the related functional assays. DCA effectively suppressed migration/invasion (Fig. 5a) and sphere formation (Fig. 5b) of ALDH+CD44+ cells in vitro. We also determined cell viability of ALDH+CD44+ cells after 10 mM DCA treatment. By XTT assay and cell counting, DCA did not affect cell viability (Supplementary Fig. S4A, B). In combination with cisplatin, DCA enhanced the effects of the drug on ALDH+CD44+ SKOV3 cells by XTT (Fig. 5c) and cell counting (Supplementary Fig. S4C). Moreover, DCA suppressed *OCT4*, *KLF4*, and *BMI1* mRNA levels in the ALDH+CD44+ subset (Fig. 5d).

To assess the effects of DCA on tumour formation in vivo, we inoculated control or DCA-pre-treated ALDH+CD44+ SKOV3 cells into the flanks of nude mice and recorded tumour formation times, sizes and weights. DCA pre-treatment of cells inhibited tumour growth and prolonged tumour formation time from an average of 11 to 25 days (Fig. 5e). Additionally, tumour size and weight were significantly reduced in DCA-pre-treated mice, compared with the control group. To determine the effects of DCA on tumour-initiating properties, an in vivo limiting dilution assay was performed with serial dilutions of DCA-pre-treated ALDH+CD44+ SKOV3 cells. Tumour incidence was decreased from 80% to 20% in the 5000-cell group, and 60% to 0% in the 2500-cell group. In addition, significantly lower estimated tumour-initiating cell frequency was found in DCA-pre-treated group compared with the control mice (Fig. 5f). In view of these findings, we propose that DCA impairs CSC properties in ovarian cancer.

### PDK4 mediates ovarian CSC characteristics through STAT3/AKT/NF-κB/IL-8 signalling

**Fig. 6:**
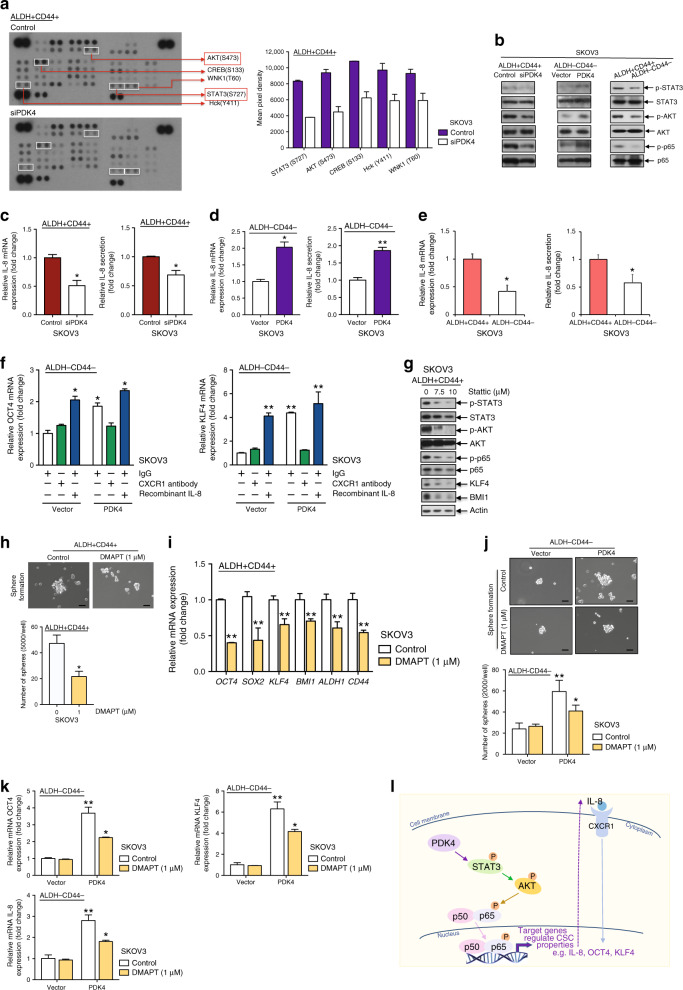
PDK4 mediates ovarian CSC characteristics through STAT3/AKT/NF-κB/IL-8 signalling. **a** (Left) Immunoblotting images and (right) quantification of deregulated phospho-kinases spotted on the phosphor-kinase array. **b** Immunoblotting showing protein expression of p-STAT3/STAT3, p-AKT/AKT and p-p65/p65 in (left) PDK4-suppressed ALDH+CD44+ SKOV3 cells, (middle) PDK4-overexpressing ALDH−CD44− SKOV3 cells and (right) ALDH+CD44+/ALDH−CD44− SKOV3 cells. qPCR analysis of relative IL-8 mRNA expression and ELISA analysis of relative IL-8 secretion in **c** PDK4-suppressed ALDH+CD44+ SKOV3 cells, **d** PDK4-overexpressing ALDH−CD44− SKOV3 cells and **e** ALDH+CD44+/ALDH−CD44− SKOV3 cells. **f** mRNA expression of relative stemness genes *OCT4* and *KLF4* in PDK-overexpressing ALDH−CD44− SKOV3 cells treated with IgG (2 μg/mL), CXCR1 antibody (2 μg/mL) or recombinant IL-8 (100 ng/mL) for 48 h. **g** Immunoblotting showing expression of p-STAT3/STAT3, p-AKT/AKT, p-p65/p65, KLF4 and BMI1 proteins in ALDH+CD44+ cells treated with the STAT3 inhibitor stattic (0, 7.5 and 10 μΜ) for 48 h. **h** Sphere formation assay was performed followed by imaging (scale bar, 50 μΜ) and quantification after DMAPT (1 μΜ) treatment of ALDH+CD44+ SKOV3 cells for 48 h. **i** mRNA expression of relative stemness genes, *OCT4*, *SOX2*, *KLF4*, *BMI1*, *ALDH1* and *CD44*, in ALDH+CD44+ SKOV3 cells treated with the NF-κB inhibitor, DMAPT, determined via qPCR. **j** The sphere formation assay was performed, followed by imaging (scale bar, 50 μΜ) and quantification after DMAPT (1 μΜ) treatment of PDK4-overexpressing ALDH−CD44− SKOV3 cells for 48 h. **k** Relative mRNA expression of OCT4 and KLF4 as well as IL-8 in PDK4-overexpressing ALDH−CD44− SKOV3 cells treated with DMAPT, determined via qPCR. **l** Proposed mechanism by which PDK4 mediates cancer cell stemness in ovarian cancer through STAT3/AKT/NF-κB/IL-8 signalling. Whether PDK4 has direct interaction with STAT3, leading to its phosphorylation will be examined in future studies (*p < 0.05, **p < 0.01, results represent means ± SD from three independent experiments).

Multiple pathways are associated with CSC regulation. A phosphokinase array was performed to elucidate the mechanism by which PDK4-regulated stemness in ovarian cancer using ALDH+CD44+ SKOV3 cells transiently transfected with siPDK4 or control siRNA, and ImageJ analysis was applied to quantify the intensity of the spots on the arrays. Five protein kinases (STAT3/S727, AKT/S473, CREB/S133, Hck/Y411 and WNK/T60) showed a >1.5-fold decrease in siPDK4 treatment groups (Fig. 6a). Decreased p-STAT3 S727 and p-AKT S473 protein levels were confirmed via immunoblotting (Fig. 6b). Phosphorylation at S727 is crucial to maximise STAT3 transcriptional activity, which promotes tumorigenesis in prostate cancer^19^ and chronic lymphocytic leukaemia.^20^ Because NF-κB is one of the downstream effectors of STAT3 and AKT,^21^ we additionally detected siPDK4-mediated reduction of p-p65 S536 protein expression (p-RelA) (Fig. 6b). Phosphorylation of p65 is one of the canonical NF-κB activation indicators.^22^ Conversely, increased p-STAT3 S727, p-AKT S473 and p-p65 S536 protein expression was observed in PDK4-overexpressing ALDH−CD44− SKOV3 cells (Fig. 6b). Notably, ALDH+CD44+ cells displayed higher p-STAT3, p-AKT and p-p65 protein expression relative to ALDH−CD44− cells (Fig. 6b).

siPDK4 also suppressed mRNA and protein secretion of IL-8, a well-known NF-κB downstream target,^23^ in ALDH+CD44+ cells (Fig. 6c) and tumourspheres (Supplementary Fig. S5), while overexpression of PDK4 led to the opposite effects (Fig. 6d). IL-8 mRNA and protein secretion was significantly higher in ALDH+CD44+, compared with ALDH−CD44− SKOV3 cells (Fig. 6e). IL-8 is a highly expressed chemokine in ovarian cancers and ascites associated with poor prognosis.^24^ Notably, IL-8 is reported to regulate breast CSC.^25^ To examine the effects of IL-8 on PDK4-mediated stemness, the ALDH−CD44− subset overexpressing PDK4 was treated with an anti-CXCR1 (an IL-8 receptor) neutralising antibody or recombinant human IL-8 for 48 h. PDK4 increased *OCT4* and *KLF4* mRNA expression in ALDH−CD44− cells, similar to that observed with IL-8 treatment (Fig. 6f). Furthermore, CXCR1 pre-treatment blocked OCT4 and KLF4 protein expression induced by PDK4 in ALDH−CD44− cells (Fig. 6f).

Remarkably, ALDH+CD44+ cells treated with the STAT3 inhibitor stattic (7.5, 10 μM) displayed reduced p-STAT3, p-AKT and p-p65 protein expression (Fig. 6g), suggesting that AKT and p65 are downstream effectors of STAT3. DMAPT, a potent inhibitor of NF-κB, was shown to inhibit sphere formation capacity (Fig. 6h) and stemness gene mRNA expressions (Fig. 6i). To evaluate the effects of DMAPT on PDK4-mediated stemness, ALDH−CD44− cells transiently transfected with control or PDK4 plasmids were treated with DMAPT for 48 h. We observed a significant reduction of sphere numbers (Fig. 6j) and mRNA expression of the stemness genes (Fig. 6k) in DMAPT-treated relative to control cells in the PDK4-overexpressing ALDH−CD44− cell group, while in the ALDH−CD44− cell group transfected with the vector control, DMAPT had no significant effect on sphere formation or stemness gene expression. Notably, IL-8, a well-known NF-κB downstream target, was also reduced in PDK4-overexpressing ALDH−CD44− cells after treatment with DMAPT. Our collective findings support the involvement of STAT3/AKT/NF-κB/IL-8 signalling in regulating PDK4-mediated ovarian CSCs.

## Discussion

Metastasis, chemoresistance and relapse are frequently observed in ovarian cancer despite concerted attempts to optimise treatment options. The residual CSCs subset that survives and grows after treatment contributes to the high recurrence rate of ovarian cancer. Ascites are present in advanced stages of the disease and enriched in free-floating spheroid cells.^26^ The 3D structure of cancer cell aggregates is believed to present an efficient way to enrich CSC subpopulations.^27^ These spheroid cells exhibit CSC characteristics, such as enhanced migration, invasion and sphere formation, as confirmed by our group and other researchers.^28,29^ Consistent with these findings, we showed that tumourspheres formed from ascites-derived cells express higher levels of stemness genes.

ALDH and CD44 are associated with tumorigenesis and CSC properties in ovarian cancer when used as individual CSC markers.^30,31^ In the present study, we isolated the CSC subset of ascites-derived tumour cells and cell lines with the aid of both ALDH and CD44 markers, which are overexpressed in this tumour cell population, and validated the ALDH+CD44+ subset as an ovarian CSC subpopulation for the first time to our knowledge. Apart from enhanced migratory/invasive, clonogenic and sphere-forming capacities, we observed increased chemoresistance and upregulation of stemness gene expression of this cell population in vitro. In an in vivo xenograft model, the ALDH+CD44+ subset displayed tumour-initiating and self-renewal characteristics.

CSCs favour glycolysis to OXPHOS in hepatocellular carcinoma, breast and colorectal cancers.^8^ However, limited information is available on the mechanism underlying the metabolic switch of this subpopulation in ovarian cancer. Notably, increased anaerobic glycolysis is reported in tumourspheres derived from ovarian cancer cells.^32^ Here, we demonstrated that tumourspheres and the ALDH+CD44+ subset produce higher lactate levels and this cell subpopulation is endowed with reduced OCR. Furthermore, we observed upregulation of the mitochondrial gatekeeping enzyme, PDK4, in not only ascites-derived cancer cells relative to primary ovarian tumour cells but also in tumourspheres and the ALDH+CD44+ subset. The contribution of the Warburg effect to promoting anoikis resistance and tumour metastasis through overexpression of PDK4 has been highlighted in previous studies.^33^ The collective findings support the metabolic switch from OXPHOS to glycolysis in ovarian CSCs, especially in the ALDH+CD44+ subset.

Clinically, a positive correlation was detected between PDK4 immunoreactivity and advanced tumour stages, grades and serous histological subtypes, as well as metastasis, highlighting a potential role of PDK4 in ovarian cancer progression and aggressiveness. Moreover, higher PDK4 was correlated with shorter overall and progression-free survival, implicating its utility as a novel prognostic marker for ovarian cancer.

Functionally, PDK4 induces tumour growth through regulating the KRAS signalling pathway in lung and colorectal cancers^13^ and promotes tumorigenesis in prostate cancer cells through the activation of the CREB-mediated mTORC1 signalling pathway.^34^ Tumour growth factor-β signalling is known to promote drug resistance through regulation of PDK4 expression.^35^ Furthermore, knockdown of PDK4 has been shown to reduce hypoxia-inducible factor-1α expression and subsequent vascular endothelial growth factor secretion in neuroblastoma cells.^36^ Our functional studies on manipulation of PDK4 expression in CSC and non-CSC subpopulations isolated from ascites-derived cells demonstrated that PDK4 promoted migratory/invasive, clonogenic, and sphere-forming capacities and upregulated stemness gene expression. By IHC, the presence of PDK4 in whole ovarian tumour suggested the role of PDK4 in regulating not only stemness but also cancer properties in ovarian cancer. The role of PDK4 in ovarian cancer properties will be determined in future studies.

Mechanistically, we have determined the signalling pathways by which PDK4 regulates tumorigenesis and stemness of ovarian cancer. PDK4 is reported to bind CREB in the cytoplasm, prevent its degradation and activate the mTORC1 signalling pathway in cancer cells.^34^ Moreover, PDK4 enhances vascular calcification via SMAD1/5/8 phosphorylation in both the cytoplasm and mitochondria.^37^ To clarify the mechanistic pathways by which PDK4 regulates ovarian CSCs, lysates were screened from ALDH+CD44+ SKOV3 cells treated with or without siPDK4 for the phosphokinase array. Knockdown of PDK4 suppressed STAT3/AKT/NF-κΒ activation and IL-8 expression in ALDH+CD44+ SKOV3 cells and the opposite results were obtained upon PDK4 overexpression in ALDH−CD44− cells. STAT3/NF-κΒ pathways are shown to be activated in ovarian CSCs.^38,39^ Egusquiaguirre et al.^40^ identified a STAT3 target gene, TNFRSF1A, that controls NF-κΒ transcriptional activity through nuclear localisation of p65, while another report showed that NF-κΒ-induced IL-6 contributes to STAT3 activation.^41^ Additionally, we observed that AKT and p65 are potential downstream effectors of STAT3 in ovarian CSCs. IL-8 is known to activate AKT, indicating the existence of a positive feedback loop. IL-8 enhanced stemness gene expression of ALDH−CD44− cancer cells, while CXCR1 antibody treatment exerted the opposite effect on PDK4-overexpressing ALDH−CD44− cells, suggesting a potential regulatory role of IL-8 in PDK4-modulated stemness in ovarian cancer, consistent with data obtained from a previous study supporting the utility of IL-8 as a therapeutic target to diminish CSC activity in breast cancer.^42^ Blockade of the NF-κΒ pathway by DMAPT could suppress sphere-forming capacity and stemness gene expression in ALDH+CD44+ or PDK4-overexpressing ALDH−CD44− cells. These collective findings indicate the involvement of STAT3/AKT/NF-κΒ/IL-8 signalling activation in regulating PDK4-mediated ovarian CSCs (Fig. 6l). Whether PDK4 has direct interaction with STAT3 leading to its phosphorylation will be examined in future studies.

We further evaluated the therapeutic potential of targeting PDK4 with DCA, a pan-PDK inhibitor. The potential effects of DCA has been examined in multiple cancer types.^43–45^ However, studies to date have been primarily confined to the effects of DCA on bulk tumours and not CSC subpopulations. DCA is known to redirect the metabolism of rat glioma CSCs from glycolysis to mitochondrial respiration, with no effects on rat neural stem cells.^46^ Moreover, DCA suppresses angiogenesis and induces apoptosis in CSCs of glioblastoma,^47^ and disrupts CSC properties and mitochondrial functions of CSC subpopulation of medulloblastoma^48^ and hepatocarcinoma.^49^ Our studies showed that DCA impeded sphere formation, clonogenicity and migration/invasion, as well as stemness mRNA expression of ovarian CSCs without affecting cancer cell proliferation at low doses. In addition, DCA inhibited tumorigenesis and tumour-initiating capacities in vivo. More intriguingly, DCA attenuated the effects of cisplatin on ALDH+CD44+ cancer cells, consistent with previous findings that a dual DCA–platinum drug combination could effectively overcome resistance to cisplatin in ovarian cancer.^50^

In conclusion, we have demonstrated enhanced CSC characteristics and PDK4 overexpression in ascites-derived tumour cells. PDK4 plays essential roles in endowing CSC properties and metabolic switch of the CSC subset isolated based on tumoursphere formation/CSC markers ALDH and CD44 through a mechanism involving the STAT3/AKT/NF-κΒ/IL-8 signalling pathway. This study provides important insights supporting the therapeutic potential of targeting PDK4 via its inhibitor, DCA in the CSC subset of ovarian cancer.

## Supplementary information

Supplementary Information

## Data Availability

The datasets generated and analysed during the current study are available from the corresponding author on reasonable request.
