# Supplementary material for: Ascites-derived ALDH+CD44+ tumour cell subsets endow stemness, metastasis and metabolic switch via PDK4-mediated STAT3/AKT/NF-κB/IL-8 signalling in ovarian cancer
Source: Br J Cancer. 2020 May 11;123(2):275–87. doi: 10.1038/s41416-020-0865-z (PMC7374705; doi:10.1038/s41416-020-0865-z)
Supplement: Supplementary file 1 — Supplementary Information [file 41416_2020_865_MOESM1_ESM.docx]

**Supplementary data for**

**Ascites-derived ALDH+CD44+ tumor cell subsets endow stemness, metastasis and metabolic switch properties via PDK4-mediated STAT3/AKT/NF-κB/IL-8 signaling in ovarian cancer**

**Figure S1. Rep
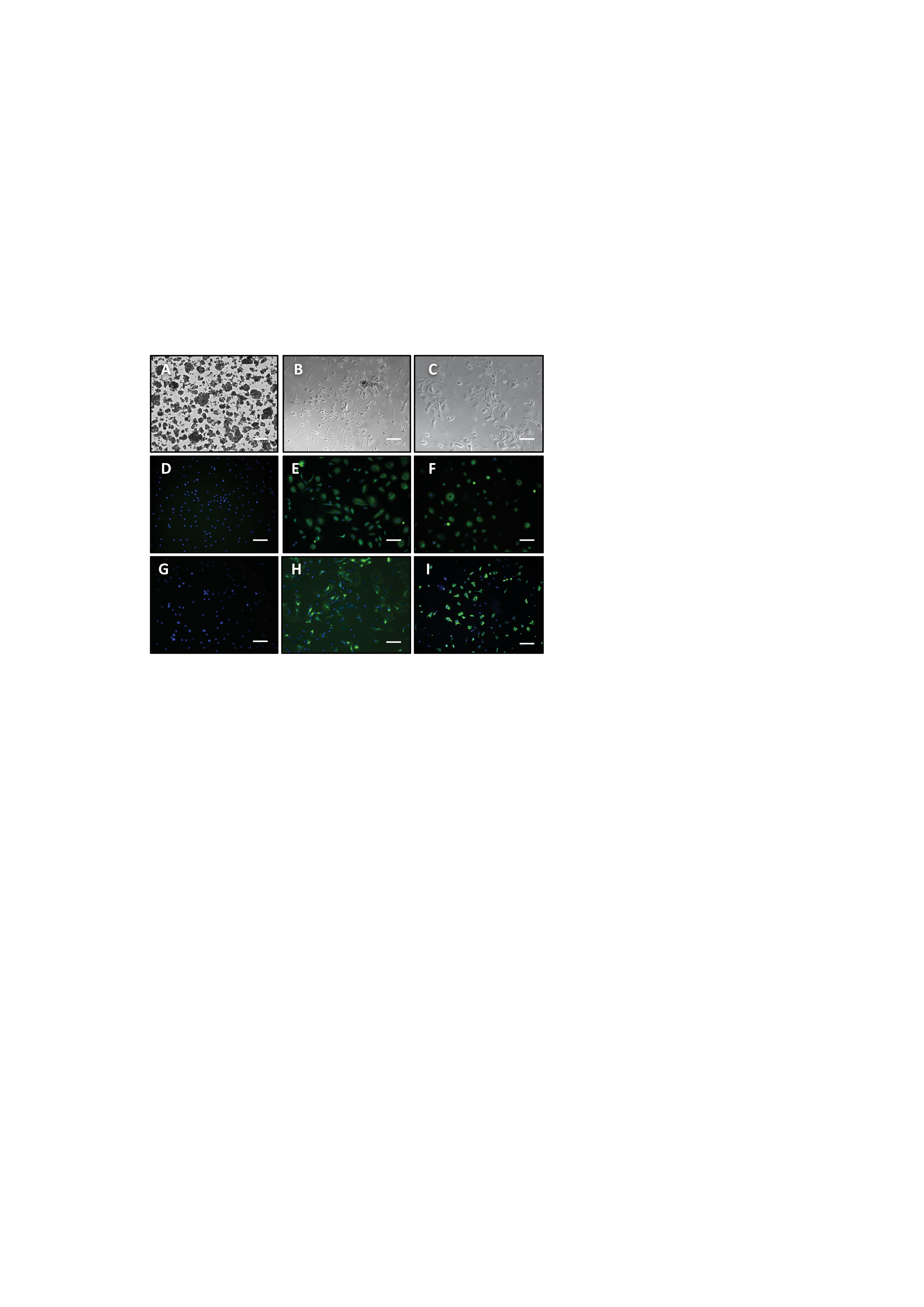
resentative images of ovarian cancer tissue samples.** (Related to Figure 1) (A) Fresh ascites (scale bar, 250 μΜ); (B) Cancer cells isolated from ascites and cultured in monolayer conditions; (C) Cancer cells isolated from primary ovarian tumors and cultured in monolayer conditions; (D-F) Immunofluoresence images showing expression of (D) CD45, (E) CK7, and (F) AE1/AE3 in cells isolated from ascites; (G-I) Immunofluoresence images showing expression of (G) CD45, (H) CK7, and (I) AE1/AE3 in cells isolated from primary tumors (scale bar, 100 μΜ).

**Figure S2. Relative cell viability of tumorspheres/ALDH+CD44+ cells transfected with control siRNA or siPDK4.** (Related to Figure 4) XTT assay on tumorspheres and ALDH+CD44+ cells derived from SKOV3 (A) and OVCAR3 (C) cells after incubation for 1 and 3 days (SKOV3), and 1 and 5 days (OVCAR3). Cell counting on tumorspheres and ALDH+CD44+ cells derived from SKOV3 and OVCAR3 cells after incubation for 3 (SKOV3; B) and 5 days (OVCAR3; D).

**
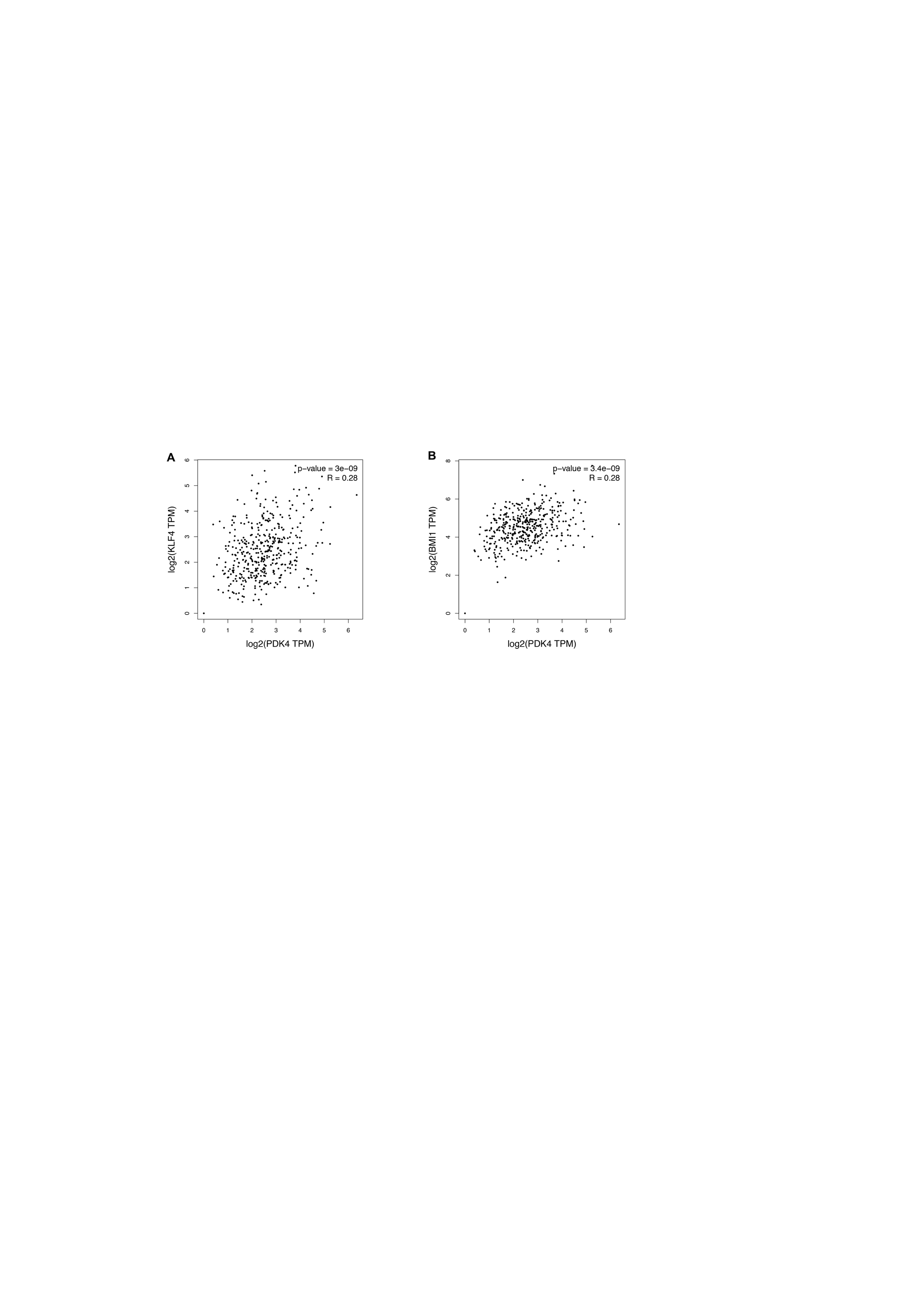
Figure S3. Correlation coefficients of PDK4 with the stemness gene set.** (Related to Figure 4) Data on PDK4 and (A) KLF4 and (B) BMI1 mRNA expression in ovarian cancer retrieved from the TGCA database. PDK4 and stem cell-related genes expression Z-scores were plotted and analyzed with GEPIA.

**Figure S4. Relative cell viability of ALDH+CD44+ SKOV3 cells after treatment with DCA or DCA plus cisplatin.** (Related to Figure 5) (A) XTT assay and (B) cell counting on ALDH+CD44+ SKOV3 cells after treatment with 10mM DCA for 3 days. (C) Cell counting on ALDH+CD44+ SKOV3 cells after treatment with 10 mM DCA plus 20 μM cisplatin for 3 days.

**Figure S5. Relative IL-8 mRNA expression (A) and secretion (B) in tumorspheres derived from SKOV3**. (Related to Figure 6) qPCR analysis of relative IL-8 mRNA expression and ELISA analysis of relative IL-8 secretion in PDK4-suppressed tumorspheres derived from SKOV3.

**Table S1. Cell lines information.**

| **Cell lines** | **Histotype** | **Derived from** |
| --- | --- | --- |
| **SKOV3** | Atypical non-serous (Clear cell/endometrioid) | Ascites |
| **OVCAR-3** | High-grade serous | Ascites |
| **OVCA420** | Serous | Primary ovarian tumor |
| **TOV112D** | Endometrioid | Primary ovarian tumor |
| **OVTOKO** | Clear cell | Metastatic spleen from ovarian tumor |

**Table S2.** Primers used for qPCR

| Genes | Primer | Sequence (5’ to 3’) |
| --- | --- | --- |
| GAPDH | Forward | TCCATGACAACTTTGGTATCGTG |
|  | Reverse | ACAGTCTTCTGGGTGGCAGTG |
| PDK4 | Forward | CCTTTGGCTGGTTTTGGTTA |
|  | Reverse | CCTGCTTGGGATACACCAGT |
| KLF4 | Forward | GGGAGAAGACACTGCGTCAA |
|  | Reverse | GGAAGTCGCTTCATGTGGGA |
| SOX2 | Forward | GGGGAAAGTAGTTTGCTGCC |
|  | Reverse | CGCCGCCGATGATTGTTATT |
| OCT4 | Forward | GGTGGAGGAAGCTGACAACA |
|  | Reverse | GTTCGCTTTCTCTTTCGGGC |
| NANOG | Forward | ATGCCTCACACGGAGACTGT |
|  | Reverse | AAGTGGGTTGTTTGCCTTTG |
| BM1 | Forward | AAATGCTGGAGAACTGGAAAG |
|  | Reverse | CTGTGGATGAGGAGACTGC |
| ALDH1 | Forward | TCCTGGTTATGGGCCTACAG |
|  | Reverse | CTGGCCCTGGTGGTAGAATA |
| CD44 | Forward | GCAAACACAACCTCTGGTCC |
|  | Reverse | CCCACACCTTCTTCGACTGT |
| IL-8 | Forward | GTGCAGTTTTGCCAAGGA |
|  | Reverse | TTATGAATTCTCAGCCCTCTTCAAAAACTTCTC |

**Table S3.** Primary antibodies used for immunoblotting.

| Protein | Animal source | Working dilution | Company | Catalog |
| --- | --- | --- | --- | --- |
| PDK4 | Rabbit | 1:600 | NOVUS | NBP1-07049 |
| KLF4 | Rabbit | 1:1000 | CST | #4038 |
| BMI1 | Rabbit | 1:1000 | CST | #58565 |
| SOX2 | Rabbit | 1:1000 | CST | #3579 |
| OCT4 | Rabbit | 1:1000 | CST | #2890 |
| p-p65 | Rabbit | 1:1000 | CST | #3033 |
| P65 | Rabbit | 1:1000 | CST | #4765 |
| p-STAT3 | Rabbit | 1:1000 | CST | #9134 |
| STAT3 | Rabbit | 1:1000 | CST | #4904 |
| p-AKT | Rabbit | 1:1000 | CST | #9271 |
| AKT | Rabbit | 1:1000 | CST | #9272 |
| Actin | Mouse | 1:50,000 | Abcam | ab6276 |

# **Materials and methods**

## Clinical samples and cell lines.

An ovarian cancer tissue microarray (TMA, OVC1021; Biomax) containing 102 cores from normal benign (5 cases) and cancer (97 cases) tissues in duplicate was used. Additionally, 16 pairs of archived formalin-fixed, paraffin-embedded (FFPE) samples from high-grade serous primary ovarian tumors and their matched metastatic foci were obtained from Queen Mary Hospital (University of Hong Kong). Fresh ovarian tumor specimens and ascitic fluid were obtained from patients with serous, clear cell and endometrioid ovarian cancer subjected to tumor-debulking surgery. To isolate single cells from tumor specimens, solid tumor tissues were finely minced with scissors and incubated with PBS (Ca^2+/^Mg^2+^-free) containing 1 mg/mL collagenase/dispase (Roche). The mixture was stirred slowly for 80 min at 37°C, filtered through sterile cell strainers (40 μm, Corning, #431750; Corning), and centrifuged at 100 g for 10 min. To obtain tumor cells from ascitic fluid samples, cell pellets were collected via centrifugation at 100 g for 10 min, and supernatant fractions discarded. Tumor cells were purified with different solutions of NaCl to exclude erythrocytes. Isolated cells from tumor specimens and ascitic fluid were resuspended in a 1:1 mixture of MCDB 105 medium and Medium 199 (Sigma-Aldrich) containing 10% fetal bovine serum (FBS; Gibco) and 1% penicillin-streptomycin (P/S). The medium was refreshed every 3 days until confluency reached 80–90%. Use of patient samples was approved by the Institution Ethical Review Board of the University of Hong Kong. The normal human ovarian surface epithelial cell line, HOSE 96-9-18, and an ovarian cancer cell line, OVCA420, were provided by Professor SW Tsao (Department of Anatomy, University of Hong Kong). Three human ovarian cancer cell lines, SKOV3, OVCAR3 and TOV112D, were purchased from ATCC (Manassas). OVTOKO was supplied by the JCRB Cell Bank (Osaka, Japan), and was purchased from CellBank Australia (Westmead, NSW, Australia). The information of ovarian cancer cell lines used in this study is shown in Table S1.

## Immunohistochemistry (IHC)

IHC staining for PDK4 was performed on TMA and FFPE-tissue sections. Briefly, after deparaffinization and rehydration, sections were subjected to 10 mM citrate sodium buffer (pH 6.0) in a microwave oven for antigen recovery and blocked in methanol containing 3% H_2_O_2_, followed by staining with primary antibody against PDK4 (Cat. #NBP1-07049, 1:100; Novus) and detection with Labelled Polymer-HRP solution (Dako) and 1% 3, 3-diaminobenzidine-hydrogen peroxide (Sigma-Aldrich). Tissue sections were analyzed using ImageScope software ([www.aperio.com](http://www.aperio.com)) with a positive pixel count algorithm. This software could be conveniently used to set the color saturation threshold, with the peak (1.0) and bottom (0) limits for the weakest and strongest positive pixel settings according to positive and negative control tissues. Positive pixel counting (positivity) was calculated automatically based on the quantity and intensity of positive pixels, which was normalized to the number of pixels counted in the selected representative areas. For each core of TMA and sample tissue section, five representative areas were selected and an average score obtained.

## Real-time PCR (qPCR)

cDNA was generated by applying total RNA extracted from ovarian cancer cells using a NucleoSpin^®^ RNA Kit (Macherey-Nagel) into SuperScript VILO™ Master Mix (Invitrogen) following the manufacturer’s instructions. Real-time PCR was conducted using synthesized cDNA as the template on an ABI Prism 7700 platform (Life Technologies) with 2×HotStart SYBR Green qPCR Master Mix (ExCell) and primers as shown in Table S2.

## Immunoblot analysis

Protein was lysed in CelLytic^TM^ M solution (Sigma-Aldrich) containing a protease inhibitor cocktail. Equal amounts of protein lysate were resolved via SDS-PAGE, followed by transfer to polyvinylidene difluoride membranes and detected with the appropriate antibodies as shown in Table S3. The human phosphor-kinase array kit (ARY003B) was obtained from R&D Systems (Minneapolis). The spot intensity on the array was quantified using ImageJ software and spot targets with >1.5-fold changes between the two groups recorded.

## ELISA assay

The culture medium collected and filtered from cancer cells was subjected to a Human IL-8 ELISA assay (Biolegend).

## Transwell migration and invasion assays

Migration and invasion were assessed using the respective assay kits (Corning). An equal quantity of cells was plated on the top chamber containing serum-free medium and migration through the 8.0 μm pore polycarbonate membrane or invasion through the Matrigel-coated membrane evaluated. After incubation for 12–24 h, migrated or invaded cells were fixed, stained, and imaged as described previously.

## Clonogenic assay

Cancer cells at a density of 500 cells/well were seeded in six-well plates containing complete medium and allowed to grow for 10 to 14 days. Surviving colonies consisting of >50 cells were counted and stained with 0.05% Crystal Violet.

## Sphere formation assay

Cancer cells were plated on an ultra-low attachment six-well plate at a density of 2000–5000 cells/well, containing CSC medium. The medium was replenished with 300 μL fresh CSC medium every second day without aspirating the old medium. After 7 to 14 days, spheres were counted and images obtained. Spheres <50 μm and individual or aggregated cells were not counted as tumorspheres.

**XTT assay and cell counting**

For the XTT assay, 2000 cells/well were seeded in 96-well plates. One, three or five days after incubation, cell viability was measured using the Cell Proliferation Kit II (Roche) in an Infinite^®^ 200 microplate reader at 492 nm (Tecan Group Ltd., Männedorf, Switzerland) according to the manufacturer’s instructions. For cell counting, 3 × 10^4^ cells were seeded in 12- or 6-well plates, and maintained in growth media. Cell numbers were counted at days 1 (12-well culture plates), 3 or 5 (6-well culture plates) using a Luna™ automated cell counter (Logos Biosystems, Annandale, VA, USA).

## Metabolic assays

Equal volumes of medium collected from cultured cells were filtered and examined using the Lactate Colorimetric Assay Kit II (BioVision), Glycolysis Assay (Abcam), and Extracellular Oxygen Consumption Assay (Abcam). The lactate production level and oxygen consumption rate were normalized to cell number.

## The Cancer Genome Atlas Dataset (TCGA) and Gene Expression Profiling Interactive Analysis (GEPIA)

The online software GEPIA (www.gepia.cancer-pku.cn) was applied to analyze the correlation between target genes and PDK4 based on TCGA database.
